# The dietetic workforce distribution geographic atlas provides insight into the inequitable access for dietetic services for people with type 2 diabetes in Australia

**DOI:** 10.1111/1747-0080.12603

**Published:** 2020-01-19

**Authors:** George Siopis, Alexandra Jones, Margaret Allman‐Farinelli

**Affiliations:** ^1^ Charles Perkins Centre, School of Life and Environmental Sciences The University of Sydney Sydney New South Wales Australia; ^2^ School of Geosciences The University of Sydney Sydney New South Wales Australia

**Keywords:** diet, dietetic service provision, dietitian, geographic information systems, GIS, type 2 diabetes mellitus

## Abstract

**Aim:**

Dietetic intervention delivered by Accredited Practising Dietitians is demonstrated to improve clinical outcomes of type 2 diabetes. The aim of the present study was to assess the accessibility to dietetic intervention for people with type 2 diabetes in Australia.

**Methods:**

Prevalence data and dietitian workforce distribution data were sourced from Diabetes Australia and Dietitians Association of Australia, respectively. Geographical information system mapping and statistical analysis were used to compare the ratios of dietitians to people with type 2 diabetes across the states of Australia and by index of socio‐economic advantage and disadvantage in each state.

**Results:**

An inequitable distribution of the dietetic workforce and that of the people with type 2 diabetes across Australia was demonstrated. An uneven distribution of the workforce is evidenced across states when compared to the distribution of type 2 diabetes prevalence; with New South Wales having a better ratio than Victoria and South Australia. Maps and prevalence data revealed the dietetic workforce was mostly concentrated in affluent urban centres whereas the type 2 diabetes prevalence rates were higher in rural and remote areas and in areas of lower socio‐economic status.

**Conclusions:**

This research highlights the need to address the limited access to dietetic intervention for those in rural, remote and disadvantaged areas which also have the greatest need. The financial burden of treating diabetic complications on the national health budget necessitates government initiatives. These should include better use of telehealth dietetic consultations and incentives for dietitians to work in rural, remote and disadvantaged areas.

## 1 INTRODUCTION

Type 2 diabetes mellitus (T2DM) has emerged as the biggest global chronic epidemic, affecting an estimated half a billion people, with a global cost of about $1.3 trillion and numbers on the rise.^1^, ^2^, ^3^ Australian data indicate 1.2 million people with T2DM on the National Diabetes Services Scheme (NDSS) register and an annual economic burden on the national health budget close to $15 billion.^4^ These numbers are further inflated when the people at high risk of developing T2DM, known as “prediabetics” are included.^4^

Diet and exercise facilitate the management of blood glucose concentrations and achievement of optimal body composition that is, lowering of adiposity and maintenance of muscle mass. Thus they are cornerstone treatments in the management of T2DM.^5^ Changing one's eating habits is a complex undertaking that requires detailed nutrition knowledge as well as the ability to overcome social and environmental barriers and it is best managed with the professional assistance of an Accredited Practising Dietitian (APD).^6^, ^7^

Research shows better clinical outcomes, such as fasting blood glucose, cholesterol and triglyceride concentrations, are achieved when the dietary intervention is led by a dietitian.^8^, ^9^, ^10^ Even a single consultation with a dietitian improved glycosylated haemoglobin (HbA1c) levels.^11^, ^12^

An initial requirement for patients to benefit from dietetic intervention is their ability to access their services. It has been suggested by Australian and United Kingdom experts that a ratio of 1 dietitian per 300 people with diabetes mellitus is required for best practice care.^13^, ^14^ In addition to the ratio, the distribution of dietitians across the country relative to the population requiring dietetic intervention within each area is crucial. Previous reports indicate shortages of dietitians in rural and regional areas.^15^ The current research used geographical information system (GIS) mapping and statistical analysis to compare the distribution of T2DM populations to that of the dietitians across Australia, to provide insight into the access to dietetic services and assist with educated recommendations to guide future dietetic service delivery.

## 2 METHODS

The postcodes of dietitians working in private practices and public health facilities were obtained from the Dietitians Association of Australia (DAA). Registered members with DAA listed as working in public health facilities as “primary care,” “clinical dietetics,” “community nutrition,” “public health – hospital,” “public health – NGO” and those working in private practice (more than 20 hours per week and “APD only”) were included in the analysis, as these members were considered to be providing dietetic counselling.

The postcodes of people with T2DM were obtained from the 2017 NDSS dataset from Diabetes Australia. For privacy reasons the number of the NDSS registrants is not displayed if the prevalence of total diabetes is 30% or more; if the number of type 2 registrants is 10 or less; and if the population per postcode is less than 100 people. These data restrictions are implemented by the NDSS, not the researchers. The indices of socio‐economic advantaged and disadvantaged postcodes were derived from the 2016 Australian Bureau of Statistics (ABS) Socio‐Economic Indexes for Area (SEIFA).^16^

ESRI ArcGIS© version 10.5 (2016) was used to create maps of the distribution of people with T2DM as well as that of the dietetic workforce available to be counselling people with T2DM across Australia. It should be noted that this involves all dietitians available for dietetic intervention and it is not a measure of those who treat people with diabetes. Further analysis was undertaken in the country's largest city, Sydney, to further expand our knowledge of dietetic workforce distribution vs that of people with T2DM.

All statistical analyses were conducted in IBM SPSS Statistics© version 24 (2016). The ratio of dietitians to people with T2DM for each postcode across Australia was calculated. For each state, the mean ratio (±SD) was calculated. The ratio in each state was compared using ANOVA with post hoc Tukey HSD test to locate differences. To test for differences between advantaged and disadvantaged socioeconomic areas, the postcodes categorised by the ABS as SEIFA deciles 8‐10 (least disadvantaged) were compared with the SEIFA deciles 1‐3 (most disadvantaged), for each state and nationally. The *z* test for differences in proportions was used to locate differences between percentages of populations with T2DM in different states and socioeconomic areas.

## 3 RESULTS

The database of 3416 non‐private practice and of 1944 private practice DAA members was obtained from DAA in August 2017. The data do not indicate if the supplied postcode is place of practice or home address. Table 1 shows the breakdown of the included and excluded dietitians.

**Table 1:** Number of Dietitians Association of Australia (DAA) registered members practising in private and non‐private practice across Australia as of August 2017

DAA membership category	Private practice	Non‐private practice	Total members	Included or excluded
Total	1944	3416	5360	
Management	0	123	123	Excluded
Research and development	0	132	132	Excluded
Marketing and communication	0	85	85	Excluded
Policy regulation	0	34	34	Excluded
Food service	0	84	84	Excluded
Teaching/education	0	159	159	Excluded
Community nutrition	0	356	356	Included
Public health: corporate	0	23	23	Excluded
Public health: govt. dept./agency	0	49	49	Excluded
Public health: NGO	0	41	41	Included
Public health: primary/community care	0	20	20	Included
Public health: university	0	30	30	Excluded
Public health: hospital	0	6	6	Included
Clinical dietetics	0	1301	1301	Included
APD only	5	0	5	Included
Full time study	22	0	22	Excluded
Paid work 20 h per week or more	1912			Included
Unemployed	5	0	5	Excluded
No category	0	787	787	Excluded
Total dietitians included in analysis	1917	1724	3641	

*Note*: Dietitians included in the analysis likely to be counselling people with type 2 diabetes mellitus are indicated.

APD stands for Accredited Practising Dietitian; NGO stands for Non‐Government Organisation.

Table 2 outlines the prevalence of people with T2DM by state, the available dietetic workforce distribution and the ratios of patients to dietitians. The mean percentage of people with T2DM is 4.4%. Northern Territory and South Australia (SA) show the highest prevalence of T2DM. The mean ratio nationwide is one dietitian per 250 patients with T2DM. The range is from one dietitian per 667 cases in Tasmania to one dietitian per 154 cases in New South Wales (NSW). The ratios are significantly different between the states across the country (*P* < .001 and *F* = 3.760) with significant differences between NSW and Victoria (*P* = .004) and between NSW and SA (*P* = .004). SA has a higher prevalence of T2DM and lower ratio of dietitians. While the prevalence of T2DM in NSW and Victoria is not different, the latter has a lower number of dietitians.

**Table 2:** The distribution of the population with type 2 diabetes mellitus registered with NDSS, the number of dietitians practicing medical nutrition therapy by subcategory of public and private practice and the ratio of dietitians to people with type 2 diabetes across Australia

State or territory	Postcodes	Type 2 registrants (T2R)	ABS population	Percentage of T2R of population	Non‐private practice dietitians	Private practice dietitians	Total dietitians	Mean ratio of dietitians per 1000 T2R (SD)
Northern Territory	800‐886	13 307	258 942	5.14%^a^	21	8	29	2.73 (8.36)
New South Wales	1355‐2898	365 102	8 084 001	4.52%b,c	500	665	1165	6.47 (27.82)^1,2^
Australian Capital Territory	2900‐2914	5752	148 271	3.88%d,e	11	13	24	6.43 (8.06)
Victoria	3000‐3996	277 541	6 132 981	4.53%b,f	485	470	955	3.19 (6.05)^1^
Queensland	4000‐4895	204 239	5 046 873	4.05%^d^	417	455	872	4.58 (8.84)
South Australia	5000‐5734	92 959	1 733 991	5.36%^a^	111	121	232	2.36 (4.99)^2^
Western Australia	6000‐6955	108 680	2 814 662	3.86%^e^	149	155	304	2.78 (7.22)
Tasmania	7000‐7470	24 409	524 679	4.65%c,f	30	30	60	1.55 (3.67)
Total (National)	800‐7470	1 091 989	24 744 400	4.41%	1724	1917	3641	4.06 (15.38)

a,b,c,d,e,f States or territories NOT sharing a common alphabetical superscript have significantly different T2R proportions.

1,2 States or territories sharing a common numerical superscript have significantly different ratios of dietitians.

Table 3 displays significant differences between advantaged and disadvantaged areas across states nationwide (*P* < .001, *F* = 4.586). A pattern of better access to dietitians in advantaged postcodes can be seen for every state and territory that results from both a smaller number of people with T2DM and more dietitians in these areas. Local inequality is evident in Queensland with advantaged postcodes exhibiting significantly more dietitians that disadvantaged (*P* < .05). Other significant differences are seen between the advantaged areas of NSW and Queensland and the disadvantaged areas of Victoria, Queensland, SA, WA and Tasmania.

**Table 3:** The distribution of the population with type 2 diabetes mellitus registered with NDSS, the number of dietitians practicing medical nutrition therapy by subcategory of public and private practice and the ratio of dietitians to people with type 2 diabetes in advantaged and disadvantaged socioeconomic areas across Australia

State or territory	Postcodes	ABS 2016 index of socio‐economic disadvantage	Type 2 registrants (T2R)	ABS population	Percentage of T2R of population	Non‐private practice dietitians	Private practice dietitians	Total dietitians	Mean ratio of dietitians per 1000 T2R (SD)	Ratio of dietitians in advantaged vs disadvantaged socio‐economic deciles
NT	800‐886	Advantaged	3826	94 563	4.0^a^	10	4	14	2.06 (2.55)	14.71
		Disadvantaged	3606	73 350	4.9^c^	1	0	1	0.14 (0.48)	
NSW	1355‐2898	Advantaged	95 557	2 929 645	3.3	262	318	580	10.97 (41.01)^1–4^	2.49
		Disadvantaged	120 741	2 077 368	5.8	44	90	134	4.40 (20.61)	
ACT	2900‐2914	Advantaged	5752	148 271	3.9^a,b^	11	13	24	6.43 (8.06)	N/A
		Disadvantaged*	N/A	N/A	N/A	N/A	N/A	N/A	N/A	
Victoria	3000‐3996	Advantaged	90 565	2 621 890	3.5	287	313	600	6.04 (7.69)	4.95
		Disadvantaged	77 162	1 244 469	6.2	49	40	89	1.22 (3.62)^1^	
Queensland	4000‐4895	Advantaged	43 365	1 507 418	2.9^d^	207	254	461	12.43 (12.14)^5,8,9^	8.94
		Disadvantaged	69 596	1 279 283	5.4	43	48	91	1.39 (5.34)^2,5–7^	
SA	5000‐5734	Advantaged	18 777	496 793	3.8^b^	52	57	109	4.44 (6.26)^6^	3.19
		Disadvantaged	41 742	617 716	6.8	20	21	41	1.39 (4.03)^3^	
WA	6000‐6955	Advantaged	33 905	1 153 056	2.9^d^	87	116	203	6.53 (12.70)^7^	9.75
		Disadvantaged	19 686	397 679	5.0^c^	15	7	22	0.67 (2.77)^4,8^	
Tasmania	7000‐7470	Advantaged	2719	88 516	3.1	8	14	22	5.75 (6.56)	15.54
		Disadvantaged	14 412	272 669	5.3	7	4	11	0.37 (1.49)^9^	
Australia (National)	800‐7470	Advantaged	294 466	9 040 152	3.3	924	1089	2013	8.05 (23.05)	4.00
		Disadvantaged	346 945	5 962 534	5.8	179	210	389	2.01 (11.40)	

*Note*: Areas NOT sharing a common alphabetical superscript have significantly different T2R proportions. Areas sharing a common numerical superscript are significantly different.

Abbreviations: ACT, Australian Capital Territory; NSW, New South Wales; NT, Northern Territory; SA, South Australia; WA, Western Australia.

* Note that there are no disadvantaged postcodes in ACT.

**Figure 1:**
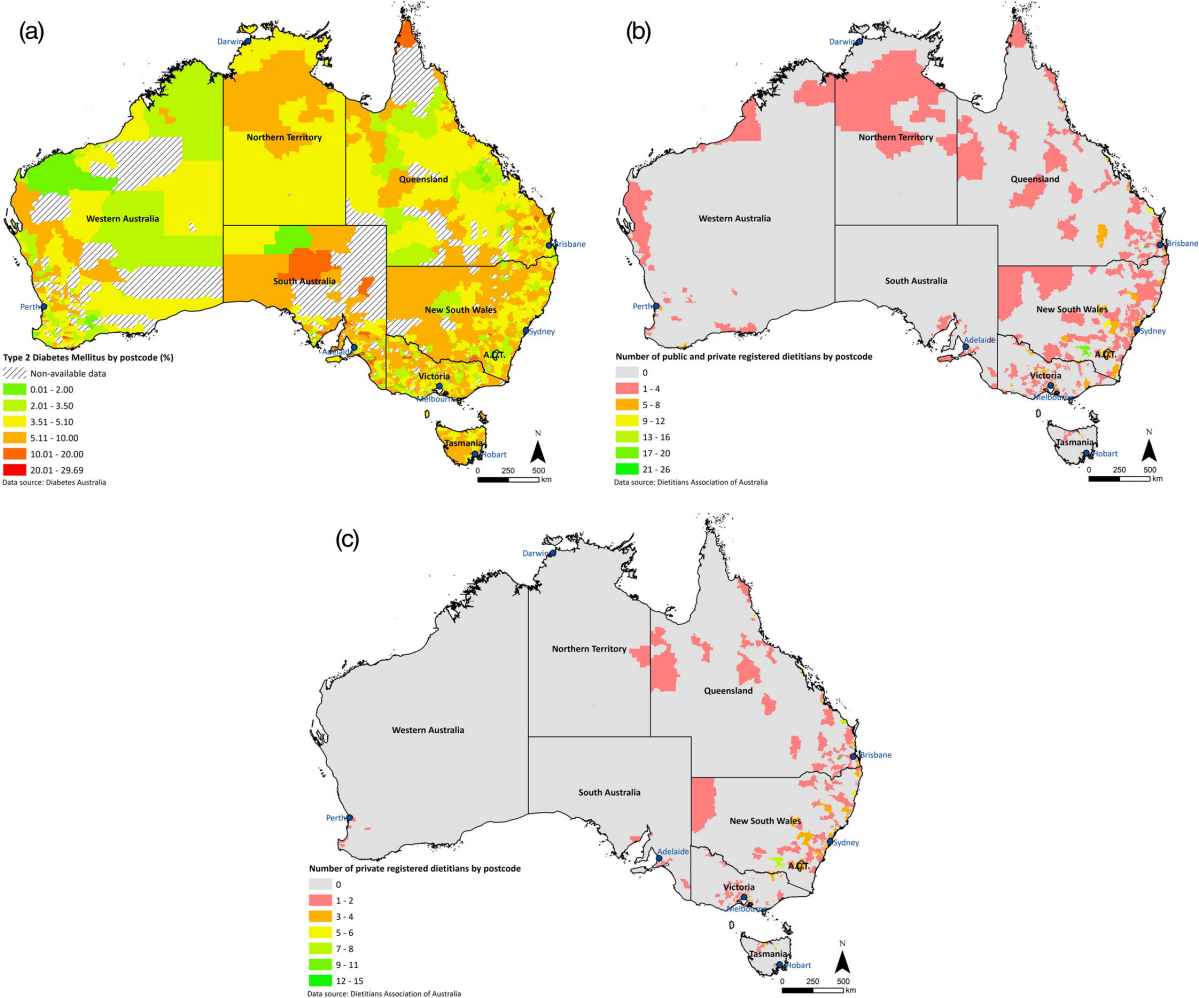
The distribution of type 2 diabetes mellitus (T2DM) and of the dietetic workforce across Australia. A, The prevalence of T2DM as a percentage of the population by postcode area across Australia. Areas that are designated as “non‐available data”, may indicate i) a prevalence greater than 30% and/or ii) less than ten people with T2DM and/or iii) a population of less than 100; and have thus been excluded from the mapping for purposes of identification. South Australia displays the highest prevalence of T2DM, followed by the Northern Territory and Tasmania. B, The distribution of clinical dietitians involved in T2DM patient counselling working in both private and public practice across Australia. C, The dietitians only working in private practice. Grey areas have zero dietitians (0), red and orange areas also designate a poor distribution of dietitians, with green shades showing the best workforce densities

Figure 1 displays the distribution of people with T2DM across Australia by postcode as well as that of the dietetic workforce providing nutritional intervention. It is evident that inland remote regions exhibit the highest prevalence of T2DM, whereas coastal urban areas have lower prevalence. On the contrary, the dietetic workforce both for public and private practice is more concentrated in the urban centres, with fewer dietitians in remote areas. The highest concentration of dietitians both in private and public sector is seen in Wagga Wagga (green area, west of ACT on map). The small green area west of Brisbane is Toowoomba.

**Figure 2:**
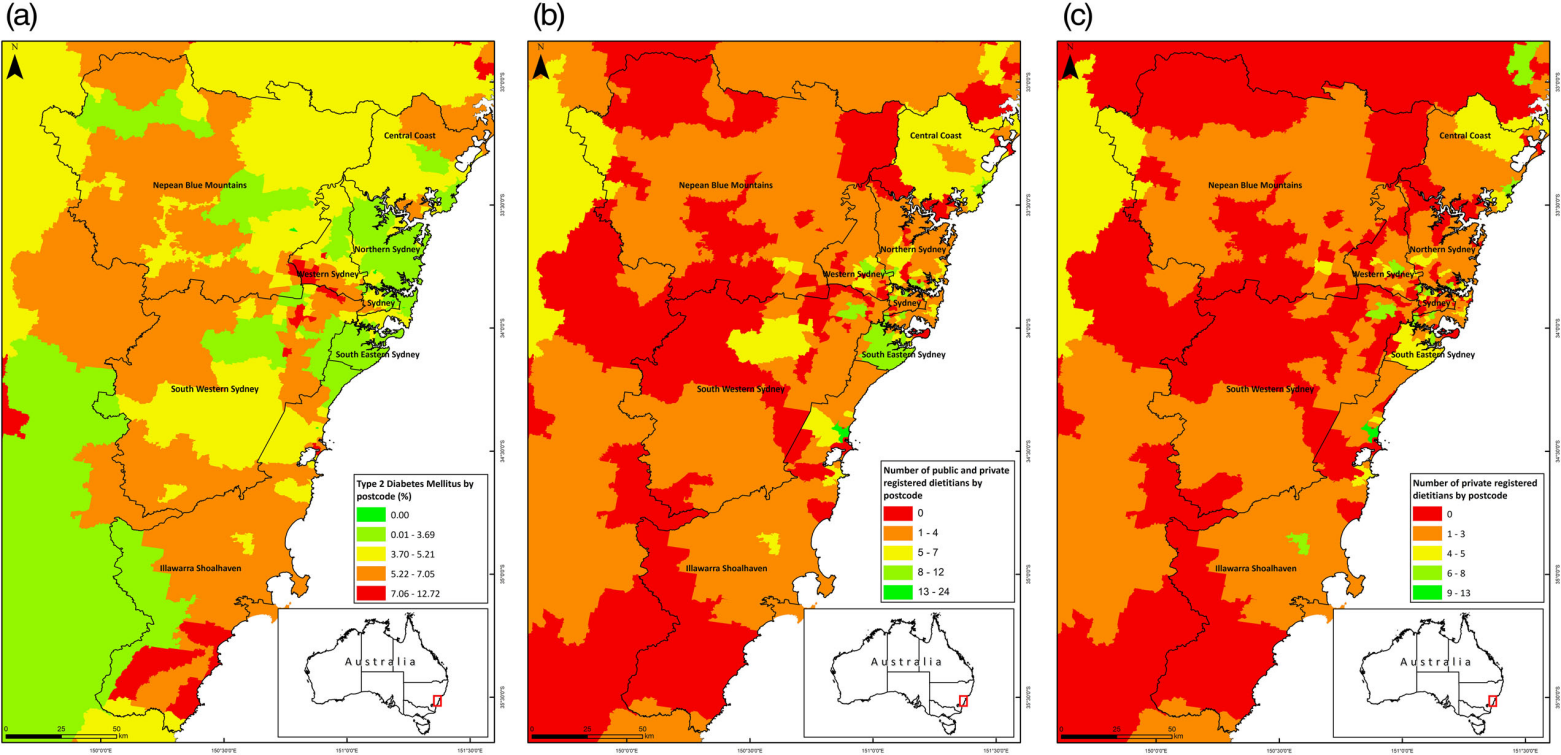
The distribution of type 2 diabetes and of the dietetic workforce in greater Sydney area. A, The prevalence of Type 2 Diabetes Mellitus (T2DM) as a percentage of the population by postcode area in greater Sydney. B,C, The distribution of the clinical dietetic work force involved in T2DM patient counselling across greater Sydney, working in both private and public practice (B) and private practice only (C). Greater Sydney area includes the following Local Health Districts (LHD): Sydney LHD, Western Sydney LHD, South Western Sydney LHD, South Eastern Sydney LHD, Northern Sydney LHD, Central Coast LHD, Illawarra Shoalhaven LHD and Nepean Blue Mountains LHD

Figure 2 examines the greater Sydney area, comprised of the health districts of Sydney, Western Sydney, South Western Sydney, South Eastern Sydney, Northern Sydney, Central Coast, Illawarra Shoalhaven and Nepean Blue Mountains. Sydney and Northern Sydney have a lower T2DM prevalence, with Illawarra Shoalhaven and Western Sydney showing a high prevalence of T2DM (see Table S1). Although, no statistically significant differences in the ratio of dietitians were detected, Western Sydney displays the lowest ratio of dietitians to people with T2DM, followed by Nepean Blue Mountains and Central Coast. In Illawarra Shoalhaven, central areas in and around Wollongong contain the majority of the dietetic workforce, whereas peripheral areas with higher T2DM prevalence have less.

## 4 DISCUSSION

The authors reported on the distribution inequality of the dietetic workforce by state and by socioeconomic areas nationally. In some cases this is driven by the higher prevalence of T2DM (eg, SA) and in others by the dietetic workforce, with the two most populous states, NSW and Victoria, exhibiting similar numbers of people with T2DM but disparity in numbers of dietitians. GIS mapping allowed differences in dietetic services between urban and rural and advantaged and disadvantaged areas to be visualised.

NSW displayed higher proportion of dietitians to people with T2DM and no disparities across the advantaged and disadvantaged areas of the Greater Sydney Area were detected using the available data. However, on a national and state‐wide basis, differences in ratios were apparent. It can only be speculated why the situation differs but it may relate to workforce retention in NSW or entry of greater numbers of graduates into the workforce. NSW may also possess more members of DAA.

In agreement with our findings, a 2012 report indicated a shortage of dietitians in Australian rural and remote areas,^15^ where one quarter of the dietetic workforce services one‐third of the population.^17^ However, the latest Australian government report on the status of the dietetics workforce in March 2014 did not raise any concerns regarding the workforce supply.^18^ That year, Health Workforce Australia (HWA) was closed and its essential functions were transferred to The Department of Health (DoH). The 2018 report by DoH on “Australia's Future Health Workforce” has no mention of the dietetics workforce because it is not a registered profession under the Australian Health Practitioner Regulation Agency.^19^

The 2015 WHO strategy report states that “the foundations for a strong and effective health workforce able to respond to the priority needs of the 21st century require matching today's supply of professionals with the demands of tomorrow's populations”.^20^ A benchmark figure of 14 dietitians per 100 000 people, based on data from Canada and the United States, was suggested in 1986.^21^ In 2006 the estimated Australian average of practising dietitians was 12.5 per 100 000 people.^22^ Our analysis indicates a current national average of 15 dietitians per 100 000 people in 2017. This does not account for the epidemic of obesity announced in 1998^23^ and the diabetes epidemic declared in 2001^24^ as well as the obesity‐epidemic‐related cancers we will realise in the next decade.^25^

The lack of dietetic services is seen globally. Dietetic shortages are reported in Spanish hospitals prompting for outsourcing of such services,^26^ as well as in Chilean hospitals, where the workforce is only about half of what is required.^27^ In Brazil, current workforce resources are not adequate, although there is a good rate of growth for the dietetic profession.^28^ Even leading countries, such as the United States, report shortages of healthcare professionals.^29^ Canadian recommendations suggest one registered dietitian for every 300‐500 patients with diabetes.^30^ The United Kingdom also presents suboptimal dietetic provision for diabetes care combined with regional inequalities.^31^, ^32^

Positive associations for health outcomes have previously been demonstrated with improved supply of healthcare services.^12^, ^33^ It has been proposed that if one in five Americans with T2DM changed their diet to reduce HbA1c by only 1% they could reverse the course of disease and save the health care system more than $10 billion a year.^34^

Australia currently scores a Gini (income equality) index of about 35.8, placing the country in a better position than most of the world, including the United States.^35^ However, the International Monetary Fund reports that Australia has one of the fastest growing income inequalities rates. With a rising trajectory of economic inequality, the inequity of access to dietetic and other health services will likely increase, as people are unable to afford to pay private practitioners and publically funded dietetic services are unable to meet the demand.

It is important to conduct further comprehensive analyses of the demand and supply of dietetic services in Australia. In the United States, the Dietetics Workforce Demand Study Task Force was appointed by the Commission of Dietetic Registration to develop a model to project supply and demand for the dietetic profession. A flexible model was produced that predicts the evolution of the workforce according to historical trends and predicted future changes.^36^

Previous reports have shown that absolute workforce number increases do not solve the problem of healthcare shortage, when the distribution of the workforce is not addressed.^37^ It is crucial to direct financial resources and secure their strategic use in correcting workforce maldistribution by for example, providing incentives for the launching of new or the relocation of some of the dietetic practices to more rural and remote areas to match the demand.

Incentives for workforce retention, in addition to those for relocation to rural areas should be considered, especially when taking into account the high cost associated with replacing allied health workers.^38^ However, effective strategies to reduce workforce turnover in rural and remote areas are missing.^39^ Telehealth and eHealth may be used to service such areas. Yet, no Medicare Benefit Schedule rebate is available for dietitians for telehealth services, although since July 2011 this is available for other allied health workers.^40^

In addition to availability, that is, the number of local services that a patient can choose from; accessibility, that is, the travel impedance (distance or time) between patient location and service location, is equally important in terms of health care service utilisation. Distance to healthcare provider as well as inadequate public transportation have been reported as barriers to healthcare access in other countries.^41^, ^42^ Travelling time has been reported as a barrier to access to services for people with diabetes in new urban areas in Melbourne, compared to established ones.^43^

The strength of the present study lies in the use of GIS mapping and the application of national data sets to examine the relationship between access to dietetic practitioners by remoteness and socioeconomic status. There are however recognised limitations. The list received from DAA on the April 24, 2017 included 5971 members. HWA's 2018 report indicated 6235 persons with a degree in nutrition and dietetics employed in various sectors across Australia.^19^ Not all dietitians working in public facilities are registered with the professional association but those in private practice wishing to have a provider number must be. Therefore, there may be an underestimation of the available dietetic workforce, however without dietetics becoming part of the Australian government health practice registration scheme, data are unavailable. Census data from the ABS was considered less appropriate as occupation is self‐reported and the only figures available were the 2011 census that reported only 2832 dietitian.^44^ Not all dietitians report workplace addresses, with some entering a residential postcode, thus the available workforce may be under‐ or over‐estimated within a given postcode. Whether the dietitians included in our mapping actually counsel people with T2DM is unconfirmed. The authors wish to recommend that future registrations with DAA include two separate fields of postcodes, in order to be able to distinguish residential and practicing locations. This should allow for multiple postcodes of employment as frequently occurs among private practitioners and principle types of dietetic interventions should be recorded. Finally, not all patients with T2DM may be identified by the NDSS. Thus both the patient numbers and dietitian numbers may be underestimated but the general direction of associations is unlikely to be changed.

This report demonstrates an uneven distribution of dietitians across the country as well as an inequitable distribution of dietitians to that of people with T2DM in Australia. This inequality is evident across states with socio‐economic advantaged areas exhibiting better ratios of distribution to disadvantaged ones. Prompt action is required considering the increasing prevalence rate for this condition, as well as the predicted increase of socio‐economic inequality in Australia. A better distribution of the dietetic workforce to allow improved access for people with T2DM should contribute towards the better management of this condition and alleviate the heavily burdened national health budget.

## CONFLICT OF INTEREST

The authors declare no potential conflict of interest.

## AUTHOR CONTRIBUTIONS

G.S. performed the statistical analyses, interpreted the data and wrote the manuscript. A.J. conducted the GIS mapping. M.A. conceived and designed the study and provided critical review of the final manuscript for submission. All authors are in agreement with the manuscript and declare that the content has not been published elsewhere.

No financial grants or other funding has been received for the present study. G.S. is a recipient of the University of Sydney International Strategic (USydIS) fund scholarship and of the Neville Whiffen scholarship. A.J. is a recipient of the University of Sydney Postgraduate Awards (UPA) scholarship. The authors wish to acknowledge the receipt of in‐kind support from Prof Greg Johnson and Mr Simon Morgan from Diabetes Australia who provided the 2014‐2015 National Health Survey T2DM data; Ms Claire Hewat who supplied DAA's membership data for a fee; and Prof Bill Pritchard who supplied the GIS computer system.

## Supporting information


**Table S1** The distribution of the population with type 2 diabetes mellitus registered with NDSS, the number of dietitians practicing medical nutrition therapy by subcategory of public and private practice and the ratio of dietitians to people with type 2 diabetes across the Local Health Districts (LHD) of the Greater Sydney Area.
