# Supplementary material for: The dietetic workforce distribution geographic atlas provides insight into the inequitable access for dietetic services for people with type 2 diabetes in Australia
Source: Nutr Diet. 2020 Jan 19;77(1):121–30. doi: 10.1111/1747-0080.12603 (PMC7383994; doi:10.1111/1747-0080.12603)
Supplement: Supplementary file 1 — Table S1 The distribution of the population with type 2 diabetes mellitus registered with NDSS, the number of dietitians practicing medical nutrition therapy by subcategory of public and private practice and the ratio of dietitians to people with type 2 diabetes across the Local Health Districts (LHD) of the Greater Sydney Area. [file NDI-77-121-s001.docx]

Supplementary Material

Table 4: The distribution of the population with type 2 diabetes mellitus registered with NDSS, the number of dietitians practicing medical nutrition therapy by subcategory of public and private practice and the ratio of dietitians to people with type 2 diabetes across the Local Health Districts (LHD) of the Greater Sydney Area.

| **Greater Sydney Area LHD** | **Type 2 Registrants (T2R)** | **ABS population** | **Percentage of T2R of population** | **Non-Private Practice Dietitians** | **Private Practice Dietitians** | **Total Dietitians** | **Mean Ratio of Dietitians per 1000 T2R (Std. Deviation)** |
| --- | --- | --- | --- | --- | --- | --- | --- |
| Sydney LHD | 17463 | 573831 | 2.95% ^a^ | 48 | 65 | 113 | 9.10 (7.32) |
| Western Sydney LHD | 43376 | 778943 | 5.26% ^b^ | 19 | 36 | 55 | 1.39 (1.56) |
| South Western Sydney LHD | 65496 | 1141214 | 5.63% | 28 | 51 | 79 | 6.38 (28.93) |
| South Eastern Sydney LHD | 27298 | 805887 | 3.30% | 54 | 96 | 150 | 6.45 (4.70) |
| Northern Sydney LHD | 31607 | 1036545 | 2.92% ^a^ | 91 | 108 | 199 | 7.81 (7.18) |
| Central Coast LHD | 16344 | 355957 | 4.57% | 14 | 26 | 40 | 4.63 (6.12) |
| Illawarra Shoalhaven LHD | 24108 | 433471 | 5.29% ^b^ | 30 | 44 | 74 | 8.14 (24.09) |
| Nepean Blue Mountains LHD | 18843 | 388501 | 4.71% | 16 | 23 | 39 | 3.97 (6.39) |
| Combined Greater Sydney Area LHDs | 244535 | 5514349 | 4.33% | 300 | 449 | 749 | 6.41 (15.23) |
| ^a,b^ LHD NOT sharing a common alphabetic superscript are significantly different in terms of T2R proportions.  Post-Hoc Tukey HSD test did not show significant differences between the LHD in terms of ratios of dietitians per T2R. | | | | | | | |
